# Rheumatoid arthritis associated cytokines and therapeutics modulate immune checkpoint receptor expression on T cells

**DOI:** 10.3389/fimmu.2025.1534462

**Published:** 2025-02-06

**Authors:** Dana Emerson, Eve Merriman, Pia P. Yachi

**Affiliations:** Immunology Discovery Research, Lilly Research Laboratories, Lilly Biotechnology Center, San Diego, CA, United States

**Keywords:** checkpoint receptor, cytokine, T cell, rheumatoid arthritis, adalimumab, tofacitinib, tocilizumab, glucocorticoid

## Abstract

**Introduction:**

We investigated the impact of rheumatoid arthritis (RA) associated cytokines and standard of care (SOC) RA therapeutics on immune checkpoint receptor (IR) expression on T cells to gain insights to disease pathology and therapeutic avenues.

**Methods:**

We assessed IR expression by flow cytometry on T cell receptor activated T cells cultured in the presence of exogenously added single cytokines or RA patient synovial fluid. We also assessed RA synovial fluid stimulated samples in the presence of various single cytokine neutralizing antibodies or SOC therapeutics, including glucocorticoids, TNF, IL-6 receptor and JAK inhibitors. In addition to IR expression, we measured the impact on cytokine secretion profiles.

**Results:**

RA-associated cytokines modulated IR expression, suggesting a role for these cytokines in regulation of disease pathology. By dissecting the influence of various inflammatory drivers within the RA inflammatory milieu, we discovered distinct regulation of IR expression by various cytokines including IL-10, IFNα/β, and TNF. Specifically, increased expression of TIM-3, PD-1, LAG-3 and CD28 in response to RA synovial fluid was driven by key cytokines including IL-6, IL-10, IL-12, IFNs, and TNF. In addition, SOC RA therapeutics such as glucocorticoids and TNF inhibitors modulated IR and cytokine expression in the presence of RA synovial fluid.

**Conclusions:**

This study points to an important and intricate relationship between cytokines and IRs in shaping immune responses in autoimmune pathology. The modulation of IR expression by RA-associated cytokines and SOC therapeutics provides new insights for the use of targeted treatments in managing RA pathology.

## Introduction

The immune system communicates with neighboring immune and stromal cells through cell surface receptors and soluble factors, such as immune checkpoint receptors (IR) and cytokines. These signals bridge context-dependent cues and specialized immune responses to balance immune cell activation, differentiation, survival, proliferation, and effector functions. T cells are key drivers of autoimmune (AI) diseases, such as rheumatoid arthritis (RA) (1–3). Cytokines guide T cell polarization into specialized effector cell subsets equipped to address different threats, such as intracellular bacteria, viruses, or extracellular dangers with distinct immune response patterns (4). This specialization is important for effective elimination of threats with minimal bystander damage to self. T cells express multiple IRs with unique signaling properties, expression patterns, and functional profiles, to enable immune response fine-tuning and homeostasis (5, 6). IRs like Programmed Death-1 (PD-1) and Cytotoxic T-lymphocyte associated protein 4 (CTLA-4) have been utilized for cancer treatment using inhibitory monoclonal antibodies (mAbs) (7). IRs also hold promise for the treatment of AI diseases through reducing inflammation and immune activity (8, 9).

The expression of many IRs, such as PD-1, T-cell immunoglobulin and mucin-domain containing 3 (TIM-3), Lymphocyte-activation gene 3 (LAG-3), and T cell immunoreceptor with Ig and ITIM domains (TIGIT), are associated with cell activation. Signaling through these receptors results in cellular inhibition via intracellular inhibitory motifs such as immunoreceptor tyrosine-based inhibitory motifs (ITIMs). Activating co-stimulatory IRs like CD28 and CD226 contribute to immune activation by promoting survival, proliferation, and effector functions through activating motifs (10, 11). IR expression is regulated by various signaling pathways, including cytokine receptor signaling. For instance, T cell receptor (TCR) activation drives the expression of PD-1, LAG-3, and TIM-3, while common γ-chain cytokines such as IL-2, IL-7, IL-15, and IL-21 further increase the expression of PD-1 and TIM-3 in T cells (12–14). IFNα has been shown to promote and sustain PD-1 transcription, while IFNβ promotes TIM-3, PD-1, and LAG-3 but inhibits the expression of other IRs like TIGIT, CD160, and B and T lymphocyte attenuator (BTLA) (14). Gaining further insights into cytokine-mediated IR expression is important for understanding disease pathology in various AI and inflammatory conditions and for optimizing therapeutic efficacy for the treatment of AI diseases.

Various signaling pathways, transcription factors, microRNAs, and post-translational modifications regulate IR expression (15). These regulatory mechanisms differ in different immune cell types and cell activation states, resulting in phenotype-selective IR regulation. Cellular phenotypic and environmental factors also play a role in regulating IR expression, allowing the immune system to balance its activation through IRs both in cell intrinsic and extrinsic manner. Multiple IRs with distinct expression patterns and downstream signaling regulation enable the immune system to orchestrate responses selectively in different cellular and tissue environments (16, 17). Therefore, it is important to study IR expression both in inflammatory environments and in a cell-specific manner.

RA is a chronic AI disorder characterized by dysregulated immune function and overexpression of cytokines such as IL-6, IL-10, IL-12, and Tumor Necrosis Factor (TNF). The interaction between immune cells and stromal cells results in characteristic symptoms of RA, including pain, swelling and progressive damage to joints. T cells play a central role in RA pathology by for instance producing inflammatory cytokines and promoting recruitment and activation of both immune and stromal cells (18). We assessed the impact of individual inflammatory cytokines and the AI disease-associated inflammatory milieu of RA synovial fluid on TCR-induced IR expression. Additionally, we evaluated the effects of various single cytokine blocking Abs and standard of care (SOC) AI therapeutics, including adalimumab (TNF Ab), prednisolone (glucocorticoid), tocilizumab (αIL-6R), and tofacitinib (JAK inhibitor), on IR expression.

This study demonstrates the dynamic nature of IR expression, influenced by the inflammatory environment, including cytokines such as IL-10, IL-12, TNF, and type I interferons, as well as RA SOC treatments. Interactions between cytokines and IRs enable unique immune responses based on the inflammatory context, suggesting that the immune system uses IRs to regulate responses in a cytokine context-dependent manner. Our findings highlight the intricate behavior of cytokines and IRs in modulating immune responses; and shed light to RA disease pathology, mechanism of action for RA SOC treatments and the potential for targeted treatments in managing RA pathology.

## Materials and methods

### Healthy donor PBMC isolation and stimulation

Leukocyte Reduction System (LRS) tubes from healthy donors (n=10) were acquired from Excellos in San Diego. PBMCs were isolated from LRS tubes using Ficoll density gradient centrifugation and stored in CryoStor CS10 freezing medium (Stemcell Technologies) in liquid nitrogen. Naïve CD4 T cells were isolated from healthy donor PBMCs using EasySep Human Naïve CD4^+^ T Cell Isolation Kit II (Stemcell Technologies), following the manufacturer’s guidelines. Isolated naïve CD4 T Cells were plated into 96 well U bottom Nuclon Delta Surface tissue culture plates (Thermo Scientific) at 50,000 cells/well. Cells were cultured in CTS OpTmizer T Cell Expansion SFM culture media (Thermo Scientific), supplemented with OpTmizer CTS T cell expansion supplement (Thermo Scientific), CTS Immune Cell SR (Gibco), GlutaMAX supplement (Gibco), and 100X antibiotic-antimycotic (Gibco). Cells were stimulated by αCD3/αCD28 magnetic Dynabeads (Gibco) at a ratio of 1:1 bead to CD4 T Cell. Additionally, cells were stimulated by indicated human recombinant cytokines (IL-1β, IL-2, IL-4, IL-6, IL-10, IL-12, IL-17, IL-23, TGFβ, IFNα, IFNβ, IFNγ) acquired from R&D Systems, each at a final concentration of 100 ng/mL. Cytokine concentration dose determined by titration to induce optimal T cell response and IR expression. Cells and supernatant were collected on day 5 for flow cytometry and MSD analysis. Cells were re-plated at 50,000 cells per well in 2 ng/ml human recombinant IL-2 until day 10. On day 10, cells were washed with media and re-stimulated with dynabeads and indicated cytokines. On day 12, cells and supernatant were collected for flow cytometry and MSD analysis. RA patient synovial fluid (n=5; mean duration of disease 11.4 years) was acquired through BioIVT. Naïve CD4 T cells were treated with 1% final concentration synovial fluid in the experiments described. Synovial fluid concentration was determined through titration to induce optimal response based on cell viability, IR expression, and cytokine expression.

### Cytokine blocking and tissue culture treatments

The cytokine blocking panel was composed of the following cytokine blocking Abs: anti-IL-1β (H1b-27, BioLegend), anti-IL-2 (MQ1-17H12, eBioscience), anti-IL-4 (8D4-8, eBioscience), anti-IL-6R (Tocilizumab similar, BioXCell), anti-IL-10 (JES3-9D7, eBioscience), anti-IL-12 (20C2, Invitrogen), anti-IL-13 (JES10-5A2, BioLegend), anti-IL-15 (34559, R&D Systems), anti-IL-17 (eBio64DEC17, eBioscience), anti-IL-18 (925008, R&D Systems), anti-IL-23 (HNU2319, eBioscience), anti-TNF (Adalimumab), anti-IFNαβR (AB10739, Abcam), anti-IFNβ (Polyclonal, Invitrogen), and anti-IFNγ (NIB42, Invitrogen). SOC treatments were used at the following final culture concentrations. Adalimumab 10 μg/mL, prednisolone (Sigma-Aldrich) 1 μM, Tofacitinib (Selleckchem) 20 nM, and Tocilizumab (BioXCell) 50 μg/mL. Concentrations were determined through literature review and titration to determine optimal dose.

### MSD assays

On day 12 post stimulation supernatants were collected and analyzed for cytokine expression by MSD (Meso Scale Discovery) V-plex 10 cytokine human pro-inflammatory panel for IL-1β, IL-2, IL-4, IL-6, IL-8, IL-10, IL-12p70, IL-13, IFNγ, and TNF expression. Samples were processed and run according to manufacturer protocol on a MESO SECTOR S 600MM instrument. Cat N05049A-1.

### Flow cytometry

On day 12 post stimulation, cells were collected for analysis. Cells were analyzed by flow cytometry for surface protein expression of IRs, along with phenotypic and proliferation markers using Abs for PD-1, LAG-3, TIM-3, TIGIT, CD226, CTLA-4, BTLA, and CD28. The following Abs were used: Near-IR fluorescent reactive dye (Invitrogen), anti-human CD4 (RPA-T4, BD), anti-human CD8 (RPA-T8, Biolegend), anti-human PD-1 (EH12.2H7, Biolegend), anti-human TIM-3 (7D3, BD), anti-human TIGIT (A15153G, Biolegend), anti-human CD226 (11A8, Biolegend), anti-human LAG-3 (11C3C65, biolegend), anti-human CD28 (CD28.2, Biolegend), and anti-human Ki67 (Ki67, Biolegend). Cells were washed and surface stained for 30 minutes at 4°C followed by fixation and washing. Intracellular staining was carried out according to eBioscience™ Foxp3/Transcription Factor Staining Buffer Set protocol. Data was acquired with a BD LSR 2 Fortessa instrument. Acquired FCS files were analyzed using FlowJo version 10.8.0.

### 65-plex immune monitoring panel

Human plasma from healthy donor and RA patients was analyzed for cytokine expression using Invitrogen ProcartaPlex™ Human Immune Monitoring Panel, 65-plex. Samples were processed and run according to a miniaturized Curiox system protocol. Plasma samples were acquired commercially through AllCells, LLC. Healthy patient samples (n=5), RA patient samples (n=8).

### Statistical analysis

Statistical analysis methods are provided in each figure legend. Briefly, data was analyzed with GraphPad Prism 10.0.2 software and were shown as mean ± standard deviation. Student t-tests for normally distributed data to determine significance between 2 groups as indicated. One-way ANOVA with Tukey’s multiple comparisons test was used for normally distributed data to determine significance between multiple groups as indicated. Statistical significance is indicated in the figure legend as follows *P<0.05, **P<0.01, ***P<0.001, ****P<0.0001.

## Results

### T cell IR expression is regulated by soluble cytokines during TCR stimulation

**Figure 1:**
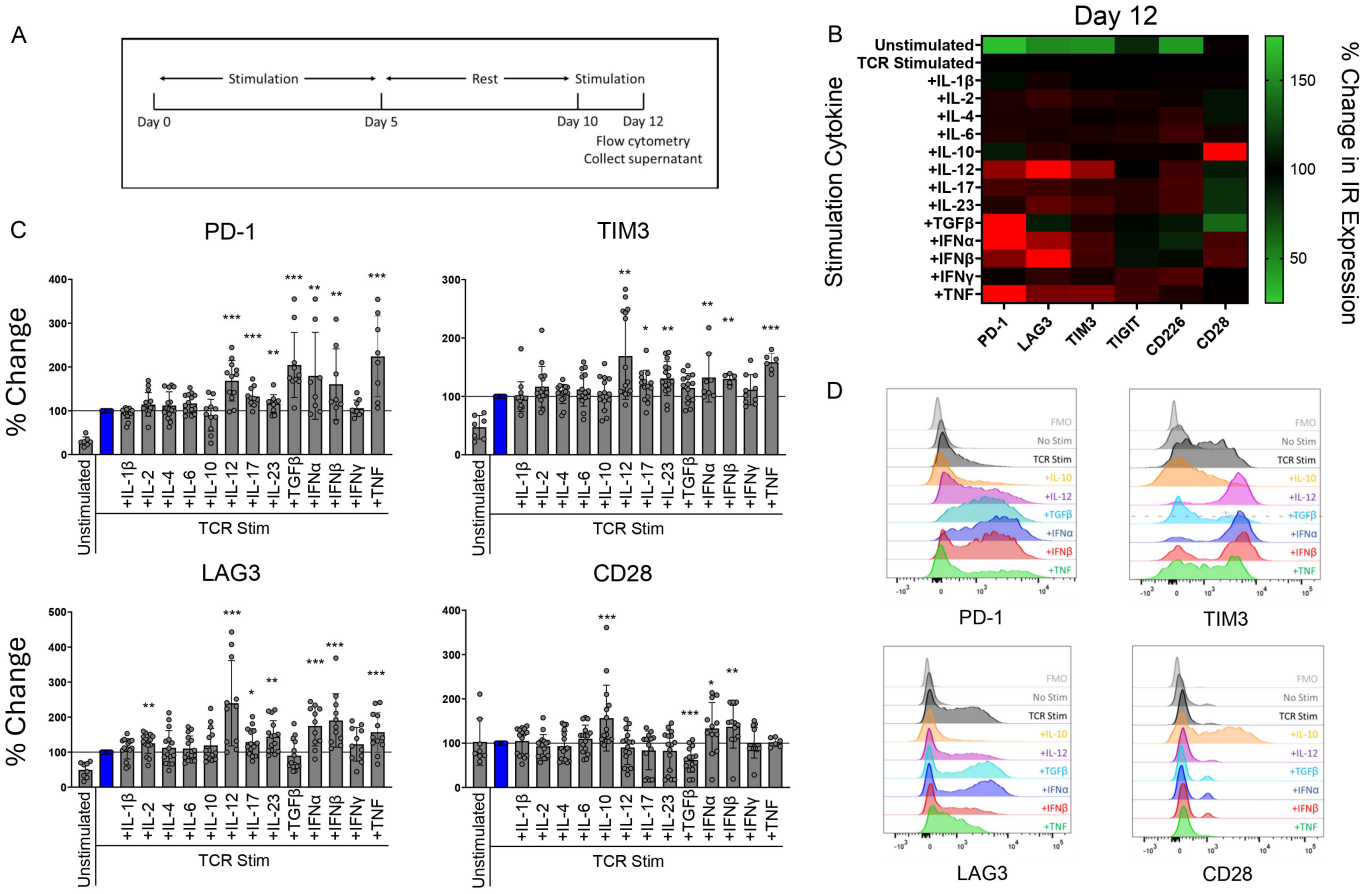
T cell IR expression is regulated by soluble cytokines during TCR stimulation. Naïve human CD4 T cells were stimulated with αCD3/αCD28 Dynabeads in the presence of indicated cytokines (100 ng/mL) for 12 days and analyzed for IR expression by flow cytometry. **(A)** treatment schematic of CD4 T cells stimulated by αCD3/αCD28 Dynabeads. **(B)** Heatmap and **(C)** bar graphs representing percent change in IR expression compared to TCR stimulation condition alone for each cytokine stimulation condition. Data is presented as mean percent change compared to TCR stimulated T cells, from 4 donors, n=2-3 per donor. **(D)** Histograms show representative PD-1, TIM-3, LAG-3, and CD28 expression in response to indicated cytokine stimulation. Statistical significance was assessed by one-way ANOVA with Tukey’s multiple comparisons test for normally distributed data. *P<0.05, **P<0.01, ***P<0.001.

We investigated the influence of various inflammatory cytokines on T cell IR expression by first isolating naïve CD4 T cells from healthy human peripheral blood mononuclear cells (PBMCs). The T cells were then TCR stimulated with αCD3/αCD28 dynabeads in the presence of various individual cytokines. On day 12 we analyzed the cells for surface IR expression by flow cytometry following the stimulation schematic (**Figure 1A**). The day 12 timepoint was chosen to mimic the chronic stimulation conditions associated with AI disease (19). Several cytokines impacted IR expression under these conditions. IL-12, IL-17, IL-23, type I interferons (IFN) significantly upregulated PD-1, LAG-3 and TIM-3 expression (**Figures 1B–D**). Type I interferons highly upregulated PD-1, and LAG-3, along with a less profound upregulation of TIM-3 and CD28. In contrast to the effects induced with type I interferons, transforming growth factor beta (TGFβ) significantly upregulated PD-1, but had no impact on TIM-3 and LAG-3 expression. Notably, TGFβ downregulated CD28 expression. CD28 expression was upregulated by IL-10 and type I IFN stimulation. IL-17, IL-23, IFNγ and TNF also upregulated TIGIT and CD226 expression. In addition, IL-4, IL-6 and IL-12 upregulated CD226, whereas IFNα negatively affected CD226 expression (**Figures 1B–D**; **Supplementary Figure 1A, B**).

Cytokine stimulation also impacted cytokine secretion from CD4 T cells (**Supplementary Figure 1C**), fitting with the ability of various cytokines to polarize T cells. For example, IL-1β, IL-2, and IL-12 enhanced the secretion of IL-6, a prominent cytokine associated with RA disease (20). These findings illustrate that key cytokines associated with AI disease uniquely and specifically impact CD4 T cell IR expression profiles and modulate T cell cytokine secretion.

### RA patients express high levels of circulating cytokines compared to healthy controls

**Figure 2:**
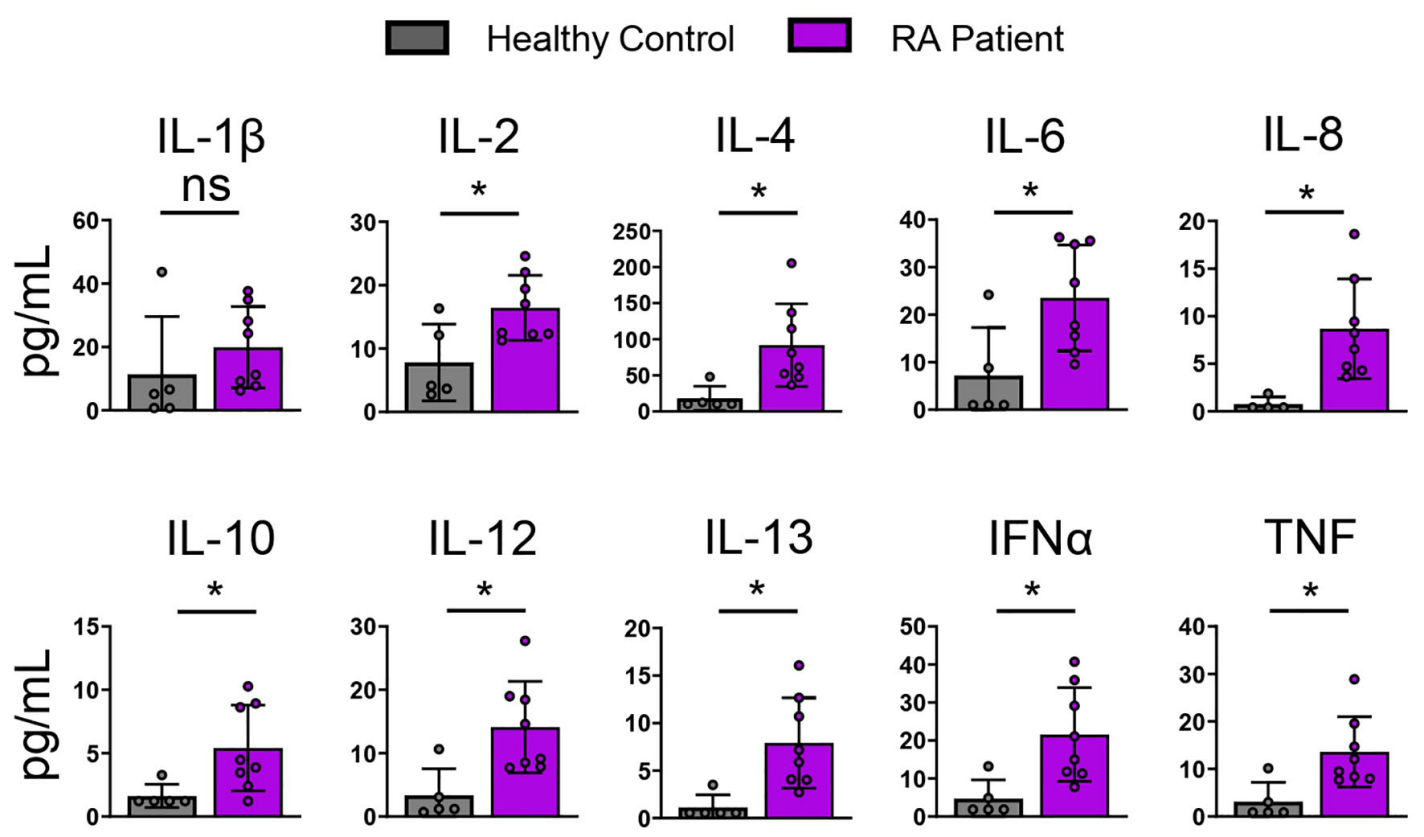
RA patients express elevated levels of circulating cytokines compared to healthy Individuals. Healthy and RA patient plasma was analyzed using a 65-plex cytokine panel. Bar graphs of selected cytokine expression is shown. Healthy control (n=5), RA patients (n=8). Statistical significance was assessed by students t-test for normally distributed data *P<0.05.

Inflammatory diseases such as RA associate with multiple inflammatory mediators (21). RA is an AI disease driven by dysregulated T and B cells and characterized by inflammation in joint synovium (22). RA patients distinguish from healthy individuals by expression of multiple inflammatory cytokines in the affected joint synovium and in systemic circulation. To characterize the systemic RA inflammatory milieu, we compared RA patient and healthy donor plasma using a 65-plex cytokine MSD panel. RA patient samples demonstrated significantly elevated levels of IFNα, TNF, IL-4, IL-6, IL-8, and IL-10 cytokines compared to the healthy controls (**Figure 2**, **Table 1**).

**Table 1:** Healthy and RA patient plasma cytokine analysis.

Cytokine	Healthy		RA		Significance	
	Mean (pg/ml)	SD	Mean (pg/ml)	SD	p-value	
LIF	4.69	3.84	16.19	5.55	0.001	**
IFN-alpha	0.71	0	6.12	3.53	0.003	**
IL-13	1.13	1.19	7.91	4.45	0.003	**
IL-18	27.71	11.37	62.65	22.06	0.003	**
IFN-gamma	4.71	4.41	21.58	11.53	0.004	**
IL-12p70	3.36	3.73	14.11	6.75	0.004	**
SDF-1alpha	51.61	56.02	254.2	134.48	0.004	**
TSLP	3.7	4.3	13.6	5.02	0.004	**
TNF-alpha	3.15	3.59	13.58	6.92	0.005	**
IL-4	18.07	14.99	91.83	53.69	0.006	**
HGF	8.69	5.12	27	13.98	0.008	**
TNF-RII	100.54	17.72	291.35	147.44	0.008	**
IL-27	12.29	19.17	75.7	51.83	0.011	*
bNGF	4.44	6.19	22.64	14.77	0.012	*
Eotaxin-3	2.23	1.51	13.07	9.08	0.012	*
IL-10	1.62	0.82	5.4	3.16	0.012	*
IL-6	7.21	9.02	23.53	10.42	0.014	*
IL-7	0.45	0.49	1.3	0.48	0.014	*
BAFF	4.26	5.75	17.75	11.12	0.015	*
IL-2	7.8	5.43	16.42	4.79	0.02	*
MIG	8.45	8.5	37.86	27.83	0.021	*
FGF-2	6.25	11.41	24.67	14.16	0.027	*
IL-5	1.32	0.72	7.77	6.55	0.027	*
SCF	6.08	3.9	11.9	3.6	0.027	*
GRO-alpha	3.81	5.19	11.65	5.3	0.028	*
Tweak	1712.31	1852.04	8317.95	6712.06	0.029	*
MCP-3	8.45	6.77	22.32	13.23	0.03	*
MIP-1 alpha	2.77	2.87	23.08	21.75	0.034	*
CD30	46.05	25.51	92.18	43.73	0.035	*
IL-2R	49.02	57.84	280.68	251.54	0.036	*
MCP-2/CCL8	2.36	1.94	4.97	1.6	0.038	*
BLC/CXCL13	34.79	33.47	80.78	32.54	0.039	*
IL-23	35.83	34.1	89.45	48.9	0.041	*
IL-20	7.42	8.21	64.23	64.69	0.042	*
MIP-1 beta	1.5	1.93	13.6	13.7	0.042	*
VEGF-A	6.48	4.17	74.38	78.2	0.044	*
IL-17A	14.24	13.08	30.52	11.92	0.054	
IL-15	3.23	3.74	29.27	33.03	0.062	
IL-31	17.53	18.93	38.63	11.92	0.067	
MIF	19.57	7.4	177.51	205.77	0.067	
sCD40L	6.36	8.3	123.63	154.5	0.069	
GM-CSF	20.99	23.66	48.98	24.02	0.07	
I-TAC	11.42	14.24	116.41	142.98	0.077	
MCP-1	9.87	9.1	35.94	35.46	0.082	
TRAIL	6.66	8.78	49.81	59.92	0.083	
MIP-3alpha	50.33	70.45	130.82	86.32	0.096	
G-CSF	15.55	17.13	33.08	16.1	0.103	
IL-22	15.89	23.58	127.43	169.4	0.107	
M-CSF	28.36	42.45	75.87	54.12	0.108	
APRIL	211.15	181.39	1210.78	1696.77	0.141	
Fractalkine	1.29	1.85	6.94	9.6	0.145	
IL-3	36.17	65.53	181.86	243.01	0.145	
IL-1 alpha	1.34	2.14	4.83	6.26	0.181	
IL-8	3.88	6.28	8.67	4.9	0.19	
IL-21	4.77	3.99	44.62	78.06	0.192	
Eotaxin	1.53	2.64	18	35.17	0.228	
IP-10	15.31	7.67	53.98	89.15	0.261	
ENA78	50.21	80.94	2614.77	6524.8	0.303	
MMP-1	11	14.08	1192.4	3103.75	0.317	
IL-1 beta	11.38	16.31	19.91	11.99	0.347	
TNF-beta	127.67	245.83	11.35	4.81	0.35	
Eotaxin-2	12.31	23.12	21.71	18.9	0.469	
IL-16	79.33	118.4	53.43	14.9	0.651	
IL-9	15.12	16.54	17.04	7.2	0.817	
MDC/CCL22	73.3	64.49	78.71	43.44	0.874	

Healthy and RA patient plasma was analyzed using a 65-plex cytokine analysis panel. Table shows mean pg/mL, standard deviation, and p-value in protein expression between healthy individuals (n=5) and RA patient plasma samples (n=8). Statistical significance was assessed by students t-test for normally distributed data *P<0.05, **P<0.01.

### RA synovial fluid and TCR stimulated CD4 T cells upregulate PD-1, LAG-3, and TIM-3

We used RA synovial fluid to capture a disease-proximal complex inflammatory milieu. RA synovial fluid contains many of the same cytokines that are upregulated in RA patient plasma (23). We ex vivo stimulated T cells in the presence of RA synovial fluid to assess the impact of the RA soluble inflammatory milieu on CD4 T cell IR expression.

**Figure 3:**
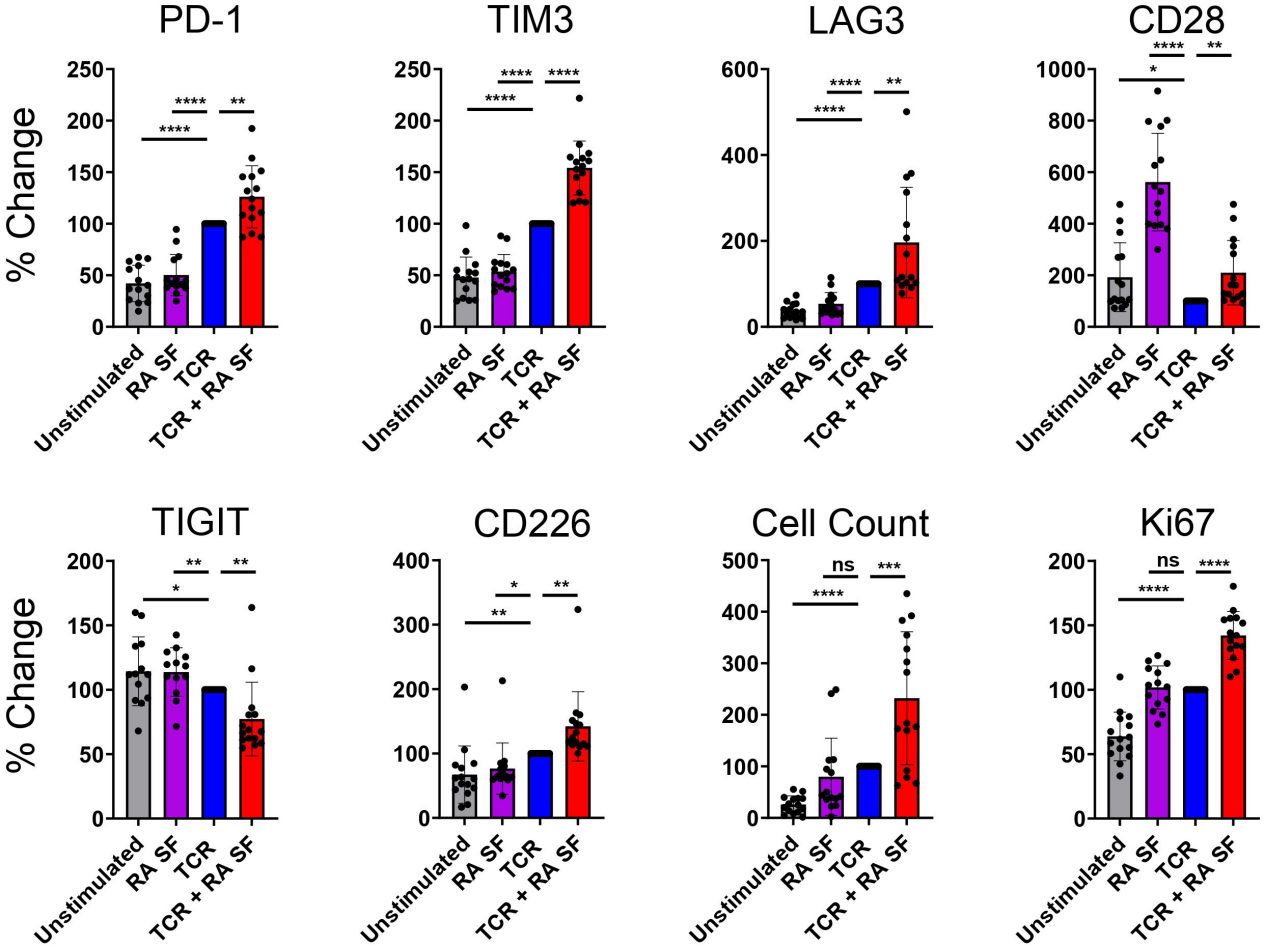
RA synovial fluid modulates CD4 T cell IR expression following TCR stimulation. Naïve human CD4 T cells were stimulated with αCD3/αCD28 Dynabeads in the presence or absence of RA synovial fluid. CD4 T cells were analyzed by flow cytometry for IR expression on day 12. Bars graphs representing percent change in IR expression compared to TCR stimulation alone based on MFI expression or cell counts. Data from 3 donors, n=5 per donor. Statistical significance was assessed by one-way ANOVA with Tukey’s multiple comparisons test for normally distributed data. *P<0.05, **P<0.01, ***P<0.001, ****P<0.0001. ns, not significant.

In the absence of TCR stimulation, RA synovial fluid had little effect on IR expression, except for upregulating CD28 expression (**Figure 3**). In the presence of TCR stimulation, RA synovial fluid enhanced TCR-induced PD-1, TIM-3, and LAG-3 expression compared to TCR stimulation alone. In addition, RA synovial fluid also enhanced the expression of the costimulatory molecules CD28 and CD226 (**Figure 3**). RA synovial fluid reduced TCR stimulation induced TIGIT expression.

These findings suggest that RA synovial fluid differentially regulates various IRs. Besides modulating IR expression, RA synovial fluid also affected cellular proliferation, as indicated by increased cell numbers and higher expression of the proliferation marker Ki67 compared to TCR stimulation alone (**Figure 3**). Furthermore, RA synovial fluid influenced the expression of cytokines in the supernatant of CD4 T cells. Specifically, RA synovial fluid increased the expression of IL-2, IL-4, IL-12, and TNF but not IL-6 compared to TCR stimulation alone. These findings further suggest that RA synovial fluid significantly modulates T cell behavior and cytokine expression (**Supplementary Figure 2**).

### Cytokine neutralization returns IR expression back to baseline in RA synovial fluid stimulated T cells

**Figure 4:**
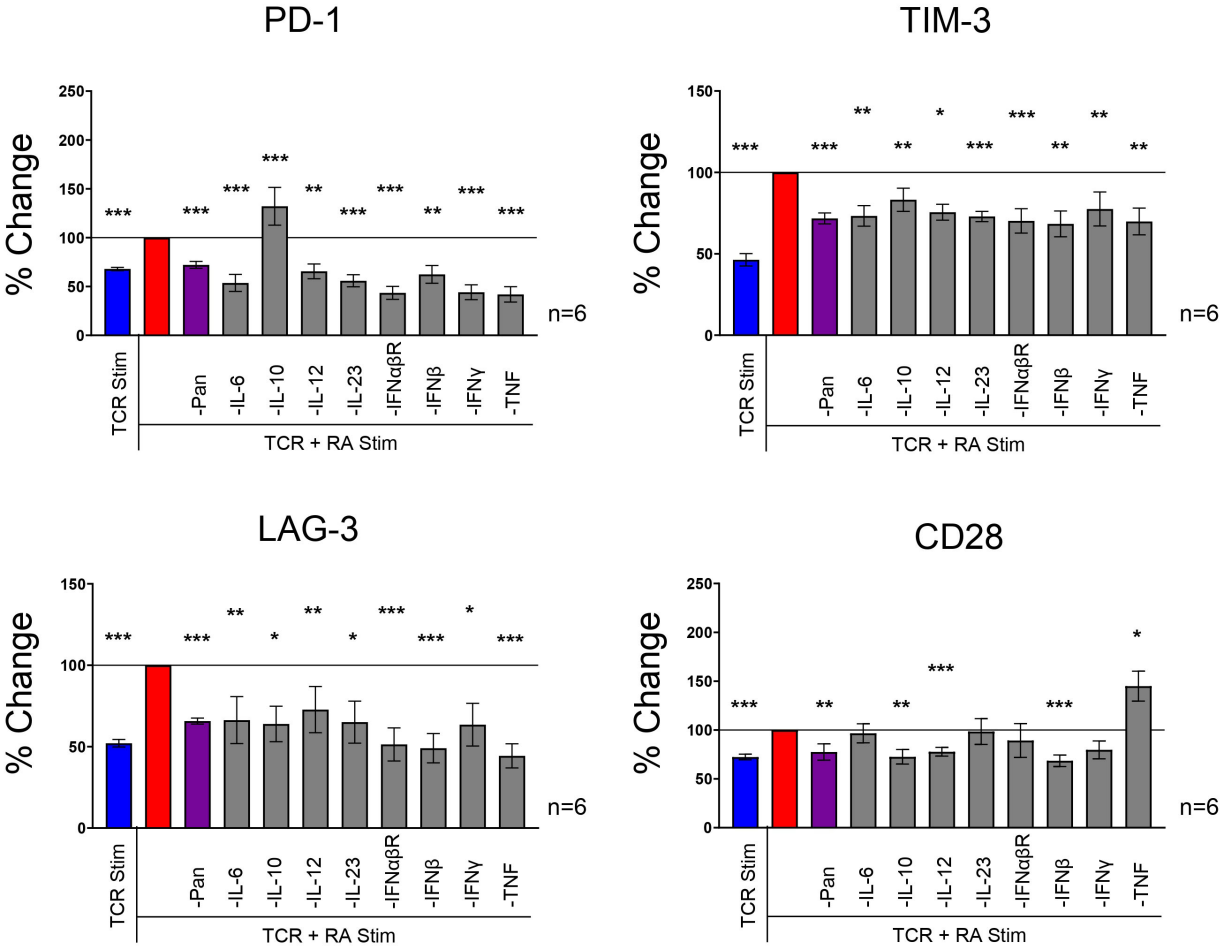
RA synovial fluid effect is neutralized with a cytokine blocking Abs. Naïve human CD4 T cells isolated from PBMCs were stimulated with αCD3/αCD28 Dynabeads in the presence or absence of RA synovial fluid or in of the combination of RA synovial fluid and a cocktail of cytokine blocking Abs, αIL-1β, αIL-2, αIL-4, αIL-6, αIL-8, αIL-10, αIL-12, αIL-13, αIL-15, αIL-17, αIL-18, αIL-23, αTNF, αIFNα, αIFNβ, and αIFNγ. CD4 T cells were analyzed by flow cytometry for IR expression on day 12. Bars graphs represent percent change in IR expression compared to TCR and RA synovial fluid stimulation based on MFI expression. Data from 3 PBMC donors and 2 synovial fluid donors (n=6). Statistical significance was assessed by one-way ANOVA with Tukey’s multiple comparisons test for normally distributed data. *P<0.05, **P<0.01, ***P<0.001.

We hypothesized that cytokines drive IR expression in the presence of RA synovial fluid based on the presence of IR modulating cytokines such as IL-10, IFNs, and TNF in RA synovial fluid. To assess the contribution of cytokines in RA synovial fluid, we neutralized several cytokines with a cocktail of blocking Abs against IL-1β, IL-2, IL-4, IL-6, IL-10, IL-12, IL-13, IL-15, IL-17, IL-18, IL-23, TNF, IFNα, IFNβ, and IFNγ. The cytokine blocking cocktail reduced PD-1, TIM-3, and LAG-3 to similar levels with TCR stimulation expression without RA synovial fluid. This suggests that cytokines in RA synovial fluid partially or fully enhance PD-1, TIM-3, and LAG-3 expression (**Figure 4**). We further observed that the cytokine blocking Ab cocktail reduced CD28 expression, suggesting that RA synovial fluid cytokines may increase CD28 expression (**Figure 4**).

Next, we individually blocked several cytokines to investigate their specific role in RA synovial fluid. We found that several individual cytokine blocking Abs, including Abs against IL-6, IL-12, IL-23, IFNs, and TNF reduced IR expression in the presence of RA synovial fluid and TCR stimulation (**Figure 4**). These findings suggest that several cytokines play a critical role in upregulating IR expression in RA synovial fluid. Several individual cytokine blocking Abs, including IL-6, IL-12, IL-23, IFNαβR, IFNβ, IFNγ, and TNF, inhibited PD-1, TIM-3, and LAG-3 expression. IL-10 neutralization inhibited TIM-3 and LAG-3 expression but enhanced PD-1 expression. Notably, blocking TNF significantly reduced PD-1, TIM-3, and LAG-3 expression but increased CD28 expression. While the blocking Ab cocktail had a negligible effect on TIGIT and CD226 expression, many individual cytokine blocking Abs enhanced TIGIT and CD226 expression (**Supplementary Figure 3**). Blocking IL-10 strongly upregulated both TIGIT and CD226. These findings suggest that multiple cytokines play important roles in driving distinct IR expression in RA synovial fluid.

### SOC RA treatments modulate IR pathways and cytokine expression

**Figure 5:**
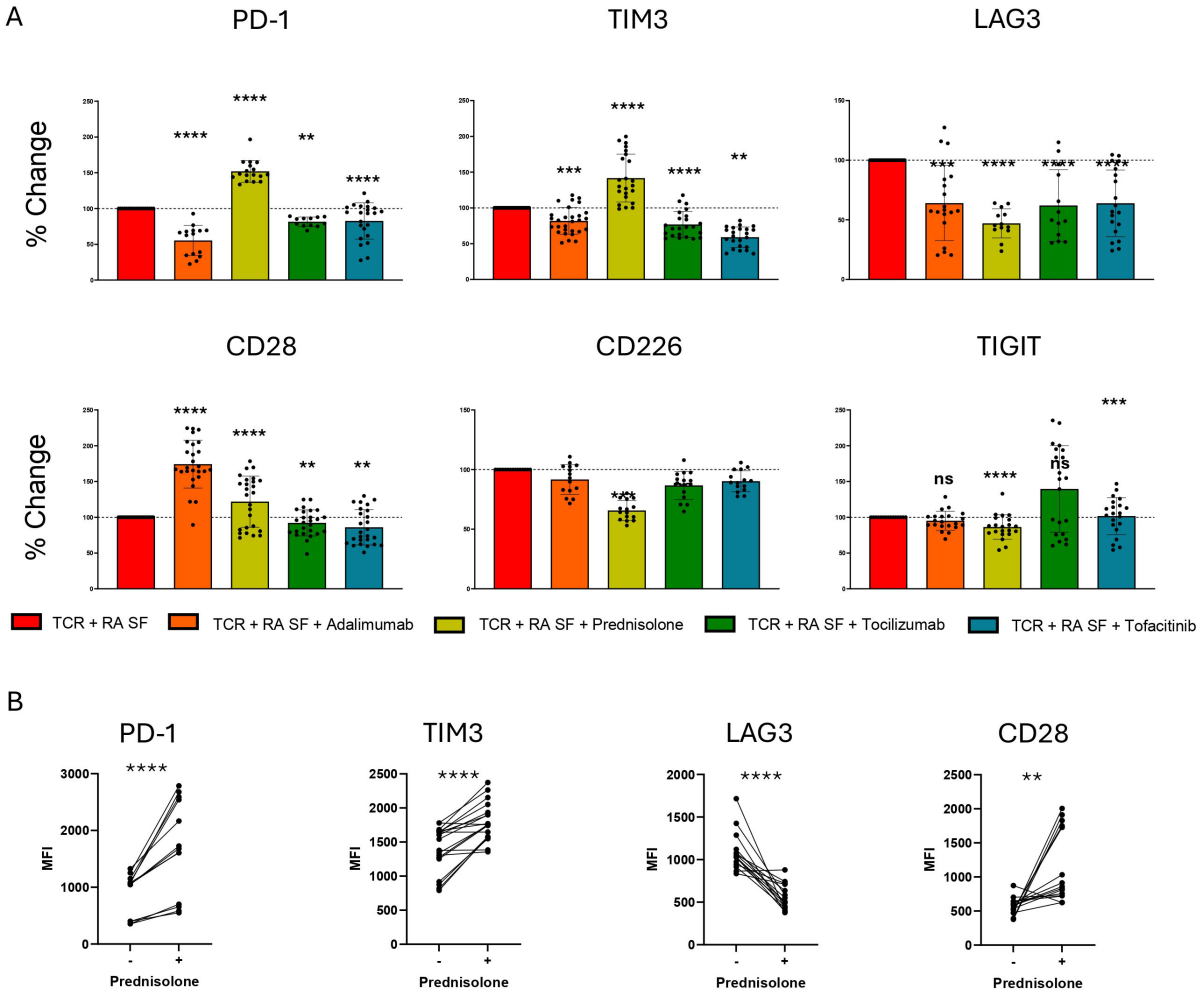
RA SOC therapeutics modulate IR expression. Naïve human CD4 T cells were stimulated with αCD3/αCD28 Dynabeads in the presence of RA synovial fluid in the presence of prednisolone 1 μM, Adalimumab 10 μg/mL, Tocilizumab 50 μg/mL or Tofacitinib 20 nM. **(A)** Bar graphs represent percent change in IR expression compared to TCR and RA synovial fluid stimulation based on MFI expression. Statistical significance was assessed by one-way ANOVA with Tukey’s multiple comparisons test for normally distributed data. **(B)** Matched individual donor IR MFI comparing TCR and RA synovial fluid stimulation with and without prednisolone treatment. Data from 3 donors, n=5 per donor. Statistical significance was assessed by students t-test for normally distributed data **P<0.01, ***P<0.001, ****P<0.0001. ns, not significant.

Recognizing the significant role of various RA synovial fluid cytokines in IR expression, we aimed to assess the role of RA therapeutics on IR and cytokine expression. Current RA therapeutics such as Glucocorticoids (GC), JAK, TNF, and IL-6 inhibitors, modulate cytokine signaling among other functions. However, their ability to modulate IR expression is less understood. Therefore, we investigated prednisolone (GC), tofacitinib (JAK 1/3 inhibitor), tocilizumab (IL-6 receptor blocking Ab), and adalimumab (TNF Ab) in the presence of TCR and RA synovial fluid stimulation. Prednisolone significantly upregulated PD-1, TIM-3 and CD28 expression in the presence of TCR and RA synovial fluid stimulation (**Figure 5A, B**, **Supplementary Figure 4A**). In contrast, prednisolone significantly downregulated LAG-3, CD226 and TIGIT expression. Prednisolone also significantly reduced expression of IL-1β, IL-2, IL-6, IL-8, IL-12, and TNF, while elevating IL-10 expression (**Supplementary Figure 4B**).

Adalimumab reduced the expression of PD-1, TIM-3, and LAG-3, but strongly upregulated CD28 expression (**Figure 5B**, **Supplementary Figure 4A**). Additionally, adalimumab reduced the expression of IL-1β, IL-2, IL-4 and IL-12, while elevating IL-10 expression (**Supplementary Figure 4B**). Tocilizumab and tofacitinib similarly reduced PD-1, TIM-3, and LAG-3 expression, with the most significant effects on LAG-3 (**Figure 5A**). Additionally, tofacitinib uniquely upregulated TIGIT expression in a proportion of donors. Tocilizumab had little effect on cytokine expression other than a slight increase in IL-10 expression. Tofacitinib reduced the expression of various cytokines, including IL-4, IL-6, IL-8, IL-10, IL-12, and TNF (**Supplementary Figure 4B**).

## Discussion

Various pathologies, including infections, cancer, and AI diseases, associate with dysregulated cytokine expression (24). IRs such as PD-1, TIM-3, LAG-3, TIGIT, CD226, and CD28 regulate and balance T cell function and immune homeostasis (25–27). We assessed the interplay between cytokine stimulation and IR expression on CD4 T cells to gain insights into T cell immune regulation. Our findings demonstrate that cytokines uniquely and specifically modulate IR expression signatures on activated CD4 T cells, suggesting that the immune system balances its activity through IRs in an inflammatory environment-specific manner. We also evaluated the impact of current RA SOC therapeutics on the interplay between cytokines and IR expression. Our findings show that SOC therapeutics elicit both pro- and anti-inflammatory consequences through IR and cytokine expression modulation. These mechanistic insights have therapeutic implications for both cytokine and emerging IR-targeting therapeutics.

PD-1 is one of the most well-characterized IRs and plays a crucial role in maintaining immune homeostasis (28). Various signaling pathways dependent on TCR, cytokine, and non-cytokine receptor signaling drive PD-1 expression (29, 30). Previous studies in mice have shown that cytokines such as IL-6, IL-12, TGFβ, and IFNα enhance PD-1 expression on activated CD8 T cells through STAT and other DNA binding elements interacting with PD-1 enhancer regions (31). However, given the lack of homology between the PDCD1 regulatory regions in mice and humans, it is important to assess cytokine effects on human PD-1 expression for accurate human translation (32). Our CD4 T cell findings demonstrated that exogenously added IL-12, IL-17, IL-23, TGFβ, IFNα/β, and TNF, but not IL-6, upregulate PD-1 expression on human T cells upon TCR activation.

Exogenous TGFβ primarily upregulated PD-1 expression with little to no effect on other IRs, including TIM-3 and LAG-3. We also identified a previously uncharacterized role for TGFβ in downregulating CD28 expression under these conditions. In contrast, IL-12 stimulation upregulated expression of multiple IRs, including PD-1, TIM-3, and LAG-3. Type I interferons upregulated PD-1, LAG-3, and CD28, with a lesser impact on TIM-3 expression. Taken together, these findings demonstrate that individual cytokines uniquely modulate IR expression profiles in CD4 T cells, suggesting that CD4 T cell activity is modulated in an inflammatory environment-specific manner.

Given the highly specialized roles of individual cytokines in triggering selective IR expression profiles, we assessed their role within the complex, disease-specific inflammatory milieu of RA synovial fluid. Like many AI diseases, RA patients exhibit upregulated cytokine expression both systemically and locally in the joint synovium compared to healthy individuals. IL-6, IL-8, IL-10, and TNF are among the most well-characterized cytokines in RA patients (29, 30). We demonstrated increased levels of IL-2, IL-4, IL-6, IL-8, IL-10, IL-12, IL-13, IFNγ, and TNF, among others, in RA patients compared to healthy individuals. These cytokines are also elevated in the RA joint synovium (23).

RA synovial fluid, in cooperation with TCR signaling, enhanced the expression of several IRs including PD-1, LAG-3, TIM-3, and CD28. Increased PD-1, LAG-3 and TIM-3 expression fitting with their inhibitory function have been associated with T cell exhaustion (33, 34). This suggests that inflammatory cytokines can serve a dual function to not only drive inflammation but to also balance immune cell activation through IR expression. Increased PD-1 and TIM-3 expression in RA synovium-derived T cells suggests that the IR modulation observed *in vitro* may also occur at the site of RA inflammation where upregulated PD-1 and TIM-3 is also observed (35). Upregulated TIM-3 expression on RA synovium-derived T cells and PBMCs has been shown to negatively correlate with disease severity (36). RA synovial fluid reduced TIGIT levels in the presence of TCR stimulation. Additionally, the paired costimulatory receptor, CD226, was upregulated. This suggests that the TIGIT/CD226 pathway may be a key dysregulated mechanism in RA, driving T cell activation. The ability of RA synovial fluid to modulate different IRs distinctly suggests that inflammatory environment can be used to specifically modulate immune responses in an environment specific manner.

We demonstrated that neutralizing multiple cytokines, including IL-6, IL-10, IL-12, IL-23, IFNαβR, IFNβ, IFNγ, and TNF in RA synovial fluid and TCR-stimulated T cells, returns the expression of IRs, such as PD-1, TIM-3, LAG-3, and CD28, to TCR-only stimulation levels. IL-6, IL-10, and TNF are commonly described cytokines in the pathogenesis of RA disease (22). Surprisingly, blocking several cytokines individually also significantly modulated IR expression. Blocking IL-10 enhanced PD-1 expression, whereas blocking TNF reduced PD-1, TIM-3, and LAG-3, and enhanced CD28 expression. The profound ability of single cytokines to modulate IR expression suggests that T cells can integrate diverse cytokine signals, requiring contributions from each of them. This suggests that T cells can integrate and proofread cytokine signals from multiple cell types and use IR expression to achieve the most appropriate self-preserving response. This also suggests that blocking individual cytokines for therapeutic purposes can alter immune cell activation balance through IR modulation.

To directly assess the effect of current RA therapeutics on IR expression, we examined the impact of TNF Ab, GC, JAKi, and IL-6R Ab using adalimumab, prednisolone, tofacitinib, and tocilizumab, respectively. Our results demonstrated that GC reduces the expression of LAG-3, TIGIT, and CD226, while increasing the expression of PD-1, TIM-3, and CD28.

The ability of GC to enhance PD-1 and TIM-3 expression was unique among the therapeutics tested. Dexamethasone has been shown to increase both mRNA and protein expression of PD-1, while LAG-3 was not upregulated (37). Additionally, our studies demonstrated that prednisolone increases IL-10 and reduces TNF expression by CD4 T cells. GC treatment has also been shown to increase IL-10 in human monocytes and in clinical settings (38). Our results extend the IL-10 effect to activated human CD4 T cells. Prednisolone treatment strongly inhibited IL-2 expression. Other GCs such as dexamethasone have also been shown to inhibit IL-2 from T cells, which is a contributing factor to GC’s anti-inflammatory activity (39). The unique property of GC to upregulate PD-1 among the SOC treatments may have both beneficial and detrimental effects due to the dual nature of PD-1 in inhibiting T cell activation and inducing T cell exhaustion.

We demonstrated that exogenous TNF stimulation combined with TCR stimulation upregulates the expression of PD-1, TIM-3, and LAG-3, whereas adalimumab in combination with TCR inhibits them. While exogenous TNF did not modulate CD28 expression upon TCR stimulation, adalimumab treatment enhanced CD28 expression in the presence of RA synovial fluid and TCR stimulation. These IR effects suggest that adalimumab may have some pro-inflammatory properties on top of its anti-inflammatory effects. Consistent with the anti-inflammatory role of adalimumab, our data showed modulation of various cytokines and significantly increased IL-10 expression. TNF inhibitors have also been shown to increase IL-10 expression from CD4 T cells in RA patients (40).

IL-6 is a prominent cytokine in RA pathogenesis, expressed at high concentrations in the RA joint synovium. IL-6 induces acute phase proteins and promotes B cell maturation, contributing to synovial inflammation (41). IL-6R primarily signals through STAT3, which is a known inducer of PD-1 expression through binding to the PD-1 promoter (40). IL-6-driven STAT3 signaling may also promote LAG-3 expression, as shown in cancer patient-derived naïve CD8 T cells (42). However, the effect of IL-6 stimulation on TIM-3 is poorly understood. Therefore, the limited effect of exogenously added IL-6 on TCR stimulation-induced PD-1, TIM-3, and LAG-3 expression was surprising. In the context of synovial fluid and TCR stimulation, blocking IL-6 or IL-6R with tocilizumab reduced the expression of PD-1, LAG-3, and TIM-3. This suggests that T cell activation-induced endogenous IL-6 is important and sufficient for the expression of various IRs, including PD-1, TIM-3, and LAG-3. IL-6 may also act to counterbalance tissue pathology by inducing the expression of inhibitory IRs.

Tofacitinib selectively targets JAK1 and JAK3 signaling, which is induced by many cytokines, including IL-2, IL-4, IL-6, IL-10, IL-12, IL-17, and IFNα/β. We demonstrated that many JAK1/3 signaling-inducing cytokines increase the expression of IRs such as PD-1, TIM-3, and LAG-3. Importantly, we showed that Tofacitinib reduces the expression of PD-1, TIM-3, LAG-3, and CD28 induced by RA synovial fluid and TCR stimulation to levels similar to TCR-only stimulation. Tofacitinib significantly reduced the expression of various cytokines, including IL-4, IL-6, IL-8, IL-10, IL-12, and TNF from activated T cells. Tofacitinib has been observed to reduce IL-10 expression in human macrophages (32). Our findings extend the effect of IL-10 reduction by Tofacitinib to human activated T cells. Tofacitinib’s ability to reduce IL-10 is unique from other therapies tested here, which all significantly increased IL-10 expression.

This study has several limitations. Plasma cytokines were analyzed from a limited number of individuals, which restricts the ability to normalize healthy vs RA samples for other variables such as age, sex and ethnicity. The use of exogenously added cytokines and RA patient synovial fluid *in vitro* may not fully replicate the complex *in vivo* environment of RA patients. The sample size of RA patient synovial fluid (n=5) was limited, which may limit the generalizability of the findings. The study also focused on a select number of cytokines, IRs and SOC therapeutics, potentially overlooking other relevant inflammatory mediators and treatments. Future studies should address these limitations by incorporating larger sample sizes, additional cytokines and IRs.

Our results shed light on the regulation of IR expression on CD4 T cells through various inflammatory cytokines and the role of individual cytokines in RA synovial fluid. Key cytokines, including IL-10, IL-12, IFNs, TGFβ, and TNF, profoundly impact the expression of PD-1, TIM-3, LAG-3, and/or CD28 on CD4 T cells. Many of these cytokines individually contribute to RA synovial fluid-induced IR expression as well. Additionally, our findings highlight the impact of current RA therapeutics on IR expression. We demonstrated that prednisolone treatment significantly increased expression of PD-1 and TIM-3, highlighting a potential for combination therapy with PD-1 and TIM-3 targeting therapeutics now advancing in the clinic. In contrast, adalimumab treatment reduced expression of PD-1, TIM-3, and LAG-3, while increasing the expression of CD28 and IL-10. These findings underscore the complex relationship between cytokines and IRs in regulating immune responses and offer insights for therapeutic interventions in AI diseases such as RA. Further study in mice and human clinical trials is warranted to investigate IR expression in response to RA therapies.

The field is still in its early stages of understanding how these sophisticated receptor systems balance immune homeostasis in both health and disease. Identifying the molecular and cellular factors associated with the disease microenvironment can drive the development of novel immunotherapies. To fully harness the potential of IR therapeutics, it is crucial to understand the cytokine expression context and the balance of these pathways in different diseases and patient subsets.

## Data Availability

The raw data supporting the conclusions of this article will be made available by the authors, without undue reservation.

## Supplementary material

Supplementary Figure 1 T cell IR expression and cytokine secretion is regulated by soluble cytokines during TCR stimulation. Naïve human CD4 T cells were stimulated with αCD3/αCD28 Dynabeads in the presence of indicated cytokines (100 ng/mL) for 12 days and analyzed for IR expression by flow cytometry and cytokine expression by MSD cytokine assay. **(A)** Bar graphs representing percent change in IR expression compared to TCR stimulation condition alone for each cytokine stimulation condition. **(B)** Histograms show representative TIGIT, and CD226 expression in response to indicated cytokine stimulation. **(C)** Heatmap representing percent change in cytokine expression compared to TCR stimulation condition alone for each stimulation condition. Cytokine expression measured by human 10-plex pro-inflammatory MSD assay. Data is presented as mean percent change compared to TCR stimulated T cells, from 4 donors, n=2-3 per donor. Statistical significance was assessed by one-way ANOVA with Tukey’s multiple comparisons test for normally distributed data. *P<0.05, **P<0.01, ***P<0.001.

Supplementary Figure 2 RA synovial fluid modulates CD4 T cell cytokine expression following TCR stimulation. Naïve human CD4 T cells were stimulated with αCD3/αCD28 Dynabeads in the presence or absence of RA synovial fluid. CD4 T cells were analyzed by MSD 10-plex human proinflammatory cytokine panel for cytokines expression. Bars graphs representing percent change in cytokine expression compared to TCR stimulation alone based on pg/mL cytokine expression. Data from 3 donors, n=5 per donor. Statistical significance was assessed by one-way ANOVA with Tukey’s multiple comparisons test for normally distributed data. *P<0.05, **P<0.01, ***P<0.001, ****P<0.0001.

Supplementary Figure 3 RA synovial fluid effect is modified by cytokine blocking Abs. Naïve human CD4 T cells isolated from PBMCs were stimulated with αCD3/αCD28 Dynabeads in the presence or absence of RA synovial fluid or in combination of RA synovial fluid and a cocktail of cytokine blocking Abs, αIL-1b, αIL-2, αIL-4, αIL-6, αIL-8, αIL-10, αIL-12, αIL-13, αIL-15, αIL-17, αIL-18, αIL-23, αTNF, αIFNα, αIFNβ, and αIFNγ. CD4 T cells were analyzed by flow cytometry for IR expression on day 12. Bar graphs represent percent change in IR expression compared to TCR and RA synovial fluid stimulation based on MFI expression. Data from 3 PBMC donors and 2 synovial fluid donors (n=6) Statistical significance was assessed by one-way ANOVA with Tukey’s multiple comparisons test for normally distributed data. *P<0.05, **P<0.01, ***P<0.001, ****P<0.0001.

Supplementary Figure 4 RA SOC therapeutics modulate IR and cytokine expression. Naïve human CD4 T cells were stimulated with αCD3/αCD28 Dynabeads in the presence of RA synovial fluid and treated with indicated therapeutics (prednisolone 1 μM, Adalimumab 10 μg/ml, Tocilizumab 50 μg/mL Tofacitinib 20 nM). **(A)** Histograms show representative PD-1, TIM-3, LAG-3, and CD28 expression in response to adalimumab and prednisolone treatment by flow cytometry. **(B)** Bar graphs represent percent change in cytokine expression compared to TCR and RA synovial fluid stimulation based on pg/mL cytokine expression measured by MSD cytokine assay. Data from 3 donors, n=5 per donor. Statistical significance was assessed by one-way ANOVA with Tukey’s multiple comparisons test for normally distributed data. *P<0.05, **P<0.01, ***P<0.001, ****P<0.0001.
