# Supplementary figures and images for: Rheumatoid arthritis associated cytokines and therapeutics modulate immune checkpoint receptor expression on T cells
Source: Front Immunol. 2025 Feb 6;16:1534462. doi: 10.3389/fimmu.2025.1534462 (PMC11840260; doi:10.3389/fimmu.2025.1534462)

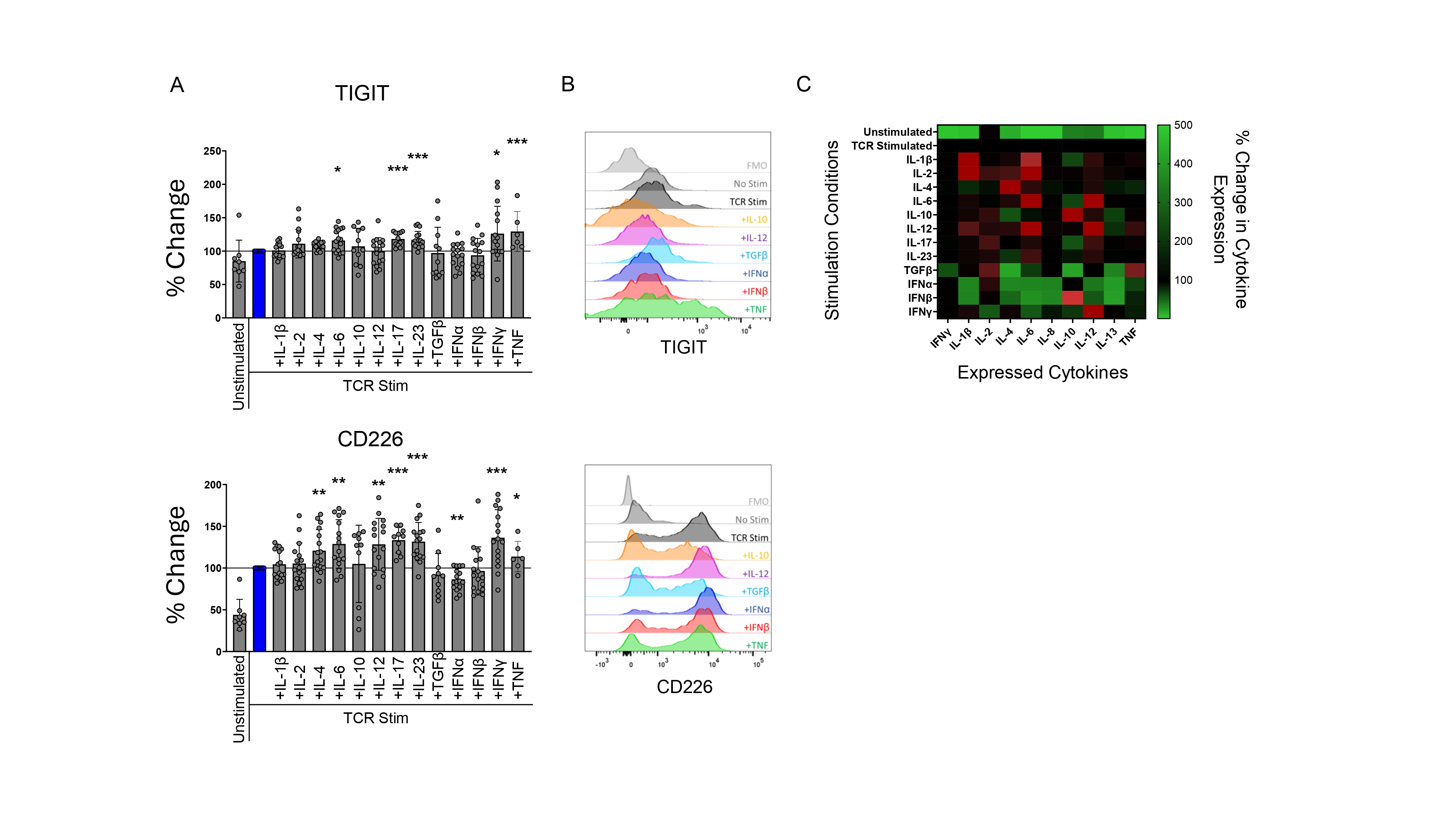

Supplement: Supplementary Figure 1 — T cell IR expression and cytokine secretion is regulated by soluble cytokines during TCR stimulation. Naïve human CD4 T cells were stimulated with αCD3/αCD28 Dynabeads in the presence of indicated cytokines (100 ng/mL) for 12 days and analyzed for IR expression by flow cytometry and cytokine expression by MSD cytokine assay. (A) Bar graphs representing percent change in IR expression compared to TCR stimulation condition alone for each cytokine stimulation condition. (B) Histograms show representative TIGIT, and CD226 expression in response to indicated cytokine stimulation. (C) Heatmap representing percent change in cytokine expression compared to TCR stimulation condition alone for each stimulation condition. Cytokine expression measured by human 10-plex pro-inflammatory MSD assay. Data is presented as mean percent change compared to TCR stimulated T cells, from 4 donors, n=2-3 per donor. Statistical significance was assessed by one-way ANOVA with Tukey’s multiple comparisons test for normally distributed data. *P<0.05, **P<0.01, ***P<0.001. [file Image1.jpeg]

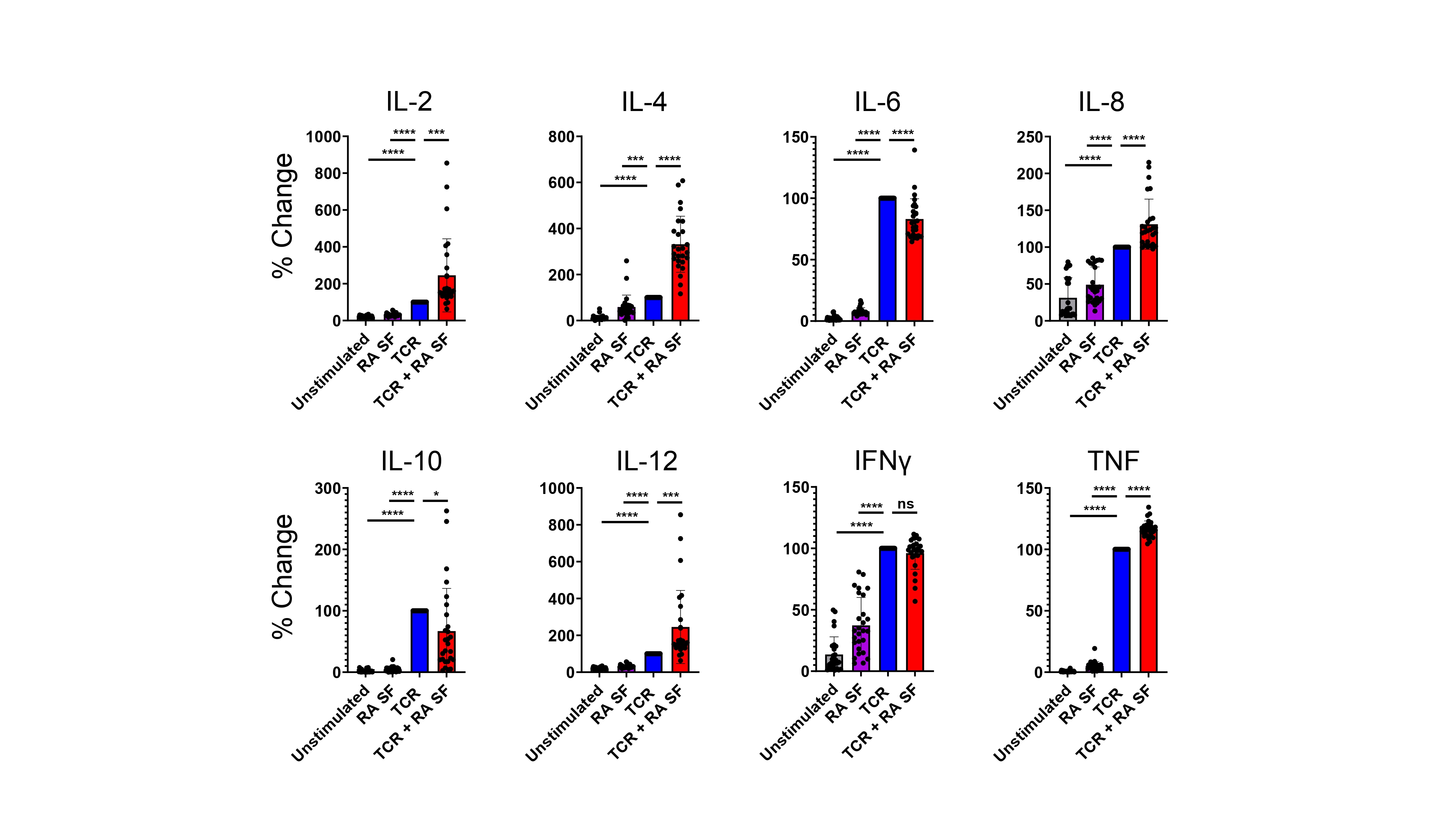

Supplement: Supplementary Figure 2 — RA synovial fluid modulates CD4 T cell cytokine expression following TCR stimulation. Naïve human CD4 T cells were stimulated with αCD3/αCD28 Dynabeads in the presence or absence of RA synovial fluid. CD4 T cells were analyzed by MSD 10-plex human proinflammatory cytokine panel for cytokines expression. Bars graphs representing percent change in cytokine expression compared to TCR stimulation alone based on pg/mL cytokine expression. Data from 3 donors, n=5 per donor. Statistical significance was assessed by one-way ANOVA with Tukey’s multiple comparisons test for normally distributed data. *P<0.05, **P<0.01, ***P<0.001, ****P<0.0001. [file Image2.jpeg]

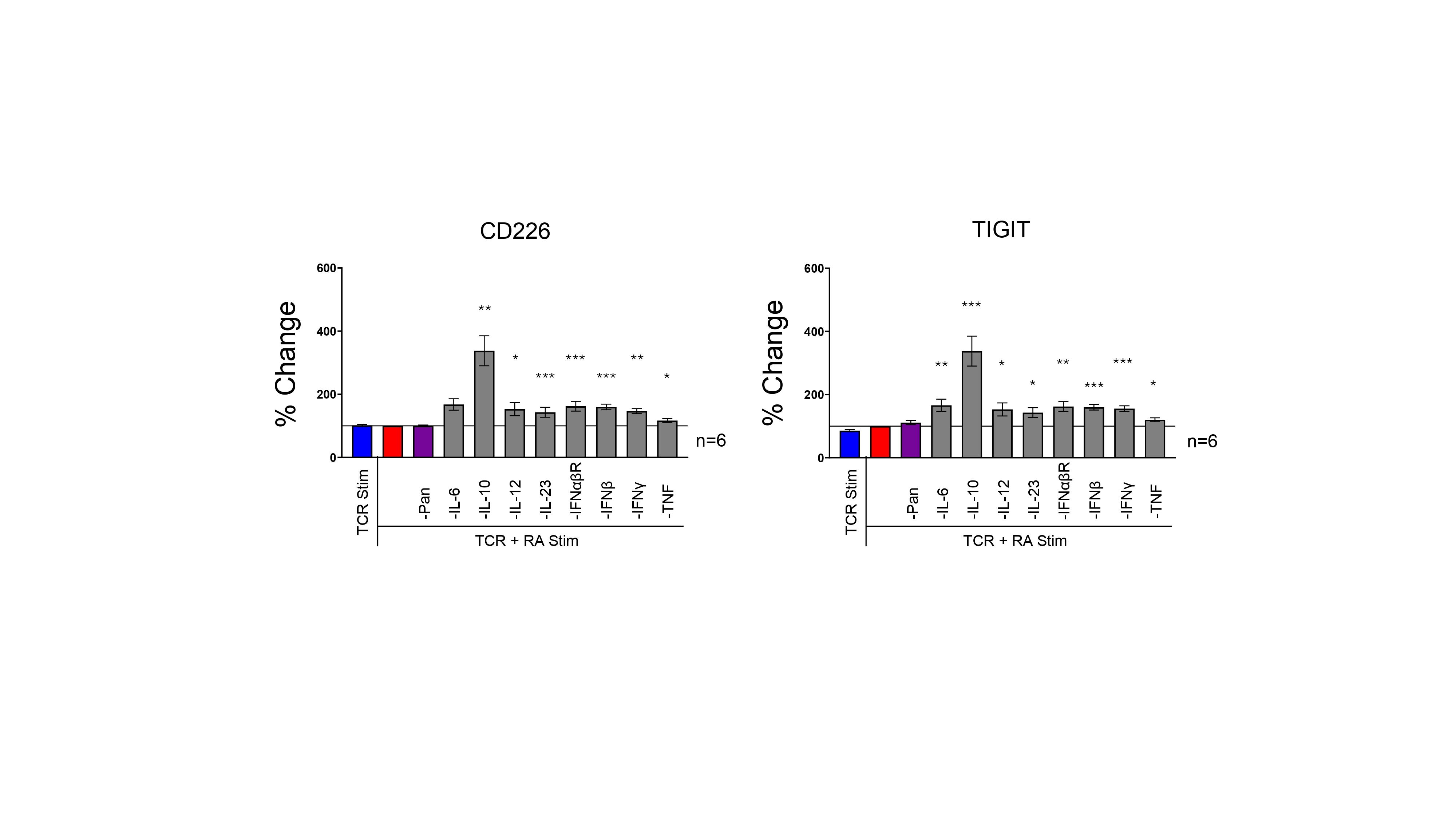

Supplement: Supplementary Figure 3 — RA synovial fluid effect is modified by cytokine blocking Abs. Naïve human CD4 T cells isolated from PBMCs were stimulated with αCD3/αCD28 Dynabeads in the presence or absence of RA synovial fluid or in combination of RA synovial fluid and a cocktail of cytokine blocking Abs, αIL-1b, αIL-2, αIL-4, αIL-6, αIL-8, αIL-10, αIL-12, αIL-13, αIL-15, αIL-17, αIL-18, αIL-23, αTNF, αIFNα, αIFNβ, and αIFNγ. CD4 T cells were analyzed by flow cytometry for IR expression on day 12. Bar graphs represent percent change in IR expression compared to TCR and RA synovial fluid stimulation based on MFI expression. Data from 3 PBMC donors and 2 synovial fluid donors (n=6) Statistical significance was assessed by one-way ANOVA with Tukey’s multiple comparisons test for normally distributed data. *P<0.05, **P<0.01, ***P<0.001, ****P<0.0001. [file Image3.jpeg]

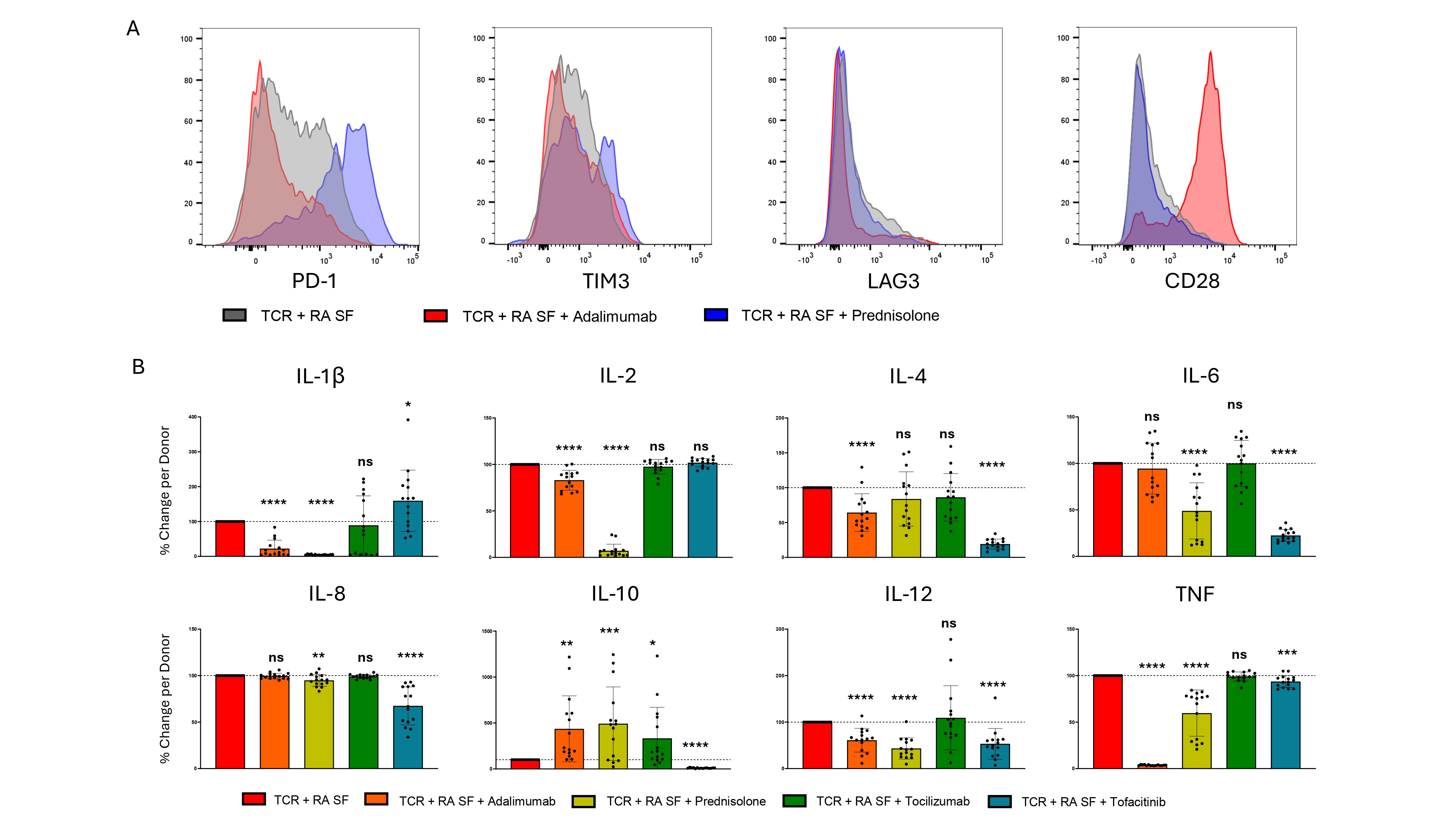

Supplement: Supplementary Figure 4 — RA SOC therapeutics modulate IR and cytokine expression. Naïve human CD4 T cells were stimulated with αCD3/αCD28 Dynabeads in the presence of RA synovial fluid and treated with indicated therapeutics (prednisolone 1 μM, Adalimumab 10 μg/ml, Tocilizumab 50 μg/mL Tofacitinib 20 nM). (A) Histograms show representative PD-1, TIM-3, LAG-3, and CD28 expression in response to adalimumab and prednisolone treatment by flow cytometry. (B) Bar graphs represent percent change in cytokine expression compared to TCR and RA synovial fluid stimulation based on pg/mL cytokine expression measured by MSD cytokine assay. Data from 3 donors, n=5 per donor. Statistical significance was assessed by one-way ANOVA with Tukey’s multiple comparisons test for normally distributed data. *P<0.05, **P<0.01, ***P<0.001, ****P<0.0001. [file Image4.jpeg]
